# Prospective Observational Study of High-Dose Carbon-Ion Radiotherapy for Pelvic Recurrence of Rectal Cancer (GUNMA 0801)

**DOI:** 10.3389/fonc.2019.00702

**Published:** 2019-07-31

**Authors:** Shintaro Shiba, Masahiko Okamoto, Hiroki Kiyohara, Tatsuya Ohno, Takuya Kaminuma, Takayuki Asao, Hitoshi Ojima, Ken Shirabe, Hiroyuki Kuwano, Takashi Nakano

**Affiliations:** ^1^Department of Radiation Oncology, Gunma University Graduate School of Medicine, Maebashi, Japan; ^2^Gunma University Heavy Ion Medical Center, Maebashi, Japan; ^3^Department of Radiation Oncology, Japanese Red Cross Maebashi Hospital, Maebashi, Japan; ^4^Big Data Center for Integrative Analysis, Gunma University Initiative for Advanced Research, Maebashi, Japan; ^5^Department of Gastroenterological Surgery, Gunma Prefectural Cancer Center, Maebashi, Japan; ^6^Department of Hepatobiliary and Pancreatic Surgery, Gunma University Graduate School of Medicine, Maebashi, Japan; ^7^Department of General Surgical Science, Gunma University Graduate School of Medicine, Maebashi, Japan

**Keywords:** carbon ion radiotherapy, rectal cancer, pelvic recurrence, prospective observational study, curative treatment

## Abstract

**Background and purpose:** Favorable clinical outcomes of carbon-ion radiotherapy for pelvic recurrence of rectal cancer have been described by previous prospective phase I/II and II studies; however, these studies were performed at a single institution. Therefore, we conducted a prospective observational study aimed at exploring whether carbon-ion radiotherapy for post-operative pelvic recurrence of rectal cancer provides a less invasive local treatment strategy with higher cure rates than other anticancer treatments.

**Materials and methods:** Patients (1) with pelvic recurrence of rectal cancer, as confirmed by histology or diagnostic imaging; (2) without distant metastasis; (3) who had undergone curative resection of their primary disease and regional lymph nodes, without gross or microscopic residual disease; and (4) with radiographically measurable tumors were included in this study. The total carbon-ion radiotherapy dose for all patients was 73.6 Gy [relative biological effectiveness (RBE)] administered in 16 fractions once daily for 4 days a week (Tuesday to Friday).

**Results:** A total of 28 patients were enrolled between October 2011 and July 2017. The median follow-up duration was 38.9 months. The 3-year overall survival, local control, and progression-free survival rates were 92, 86, and 31%, respectively. At the time of the analysis, 4 patients had local recurrence, and 7 had died of rectal cancer. None of the patients developed grade 3 or higher acute toxicities. Late toxicities occurred in 2 and 7 patients who developed grade 3 pelvic infection and grade 2 peripheral neuropathy, respectively.

**Conclusion:** Carbon-ion radiotherapy for pelvic recurrence of rectal cancer showed favorable clinical outcomes and is a highly curative and less invasive local treatment.

## Introduction

The global incidence of colorectal cancer in 2012 was an estimated 1.4 million, resulting in 693,900 deaths (1). Although surgery is the treatment of choice in patients with resectable rectal cancer, local recurrence occurs in 4–15% of patients after curative resection (2, 3). In pelvic recurrence of rectal cancer, while pelvic exenteration offers the highest potential of cure, it is highly invasive in terms of loss of function. Conventional X-ray radiotherapy (RT) for pelvic recurrence of rectal cancer is less invasive. However, the 5-year overall survival (OS) rate for treatment of pelvic recurrence of rectal cancer remains unsatisfactory at 5% to 6% after irradiation doses of 4.4–66.0 Gy (median = 30.0 Gy) (4–6).

At the Gunma University Heavy Ion Medical Center, carbon-ion (C-ion) RT was initiated in 2010 for treating various cancers, including head and neck cancer (7), non-small cell lung cancer (8), and cancers of the liver (9–11), prostate, and uterine cervix (12). Bone and soft tissue sarcomas have also been treated. C-ion RT shows higher dose localization and provides greater biological advantages than X-ray RT, including distal tail-off due to the Bragg peak, a sharp lateral penumbra, and high linear energy transfer in the Bragg peak (13–15). Compared to X-rays, C-ion beams with high linear energy transfer have a superior cell-killing effect against radioresistant tumor cells, which include hypoxic and cancer stem cells (14, 16).

Yamada et al. reported on the clinical outcomes of C-ion RT for pelvic recurrence of rectal cancer in prospective phase I/II and II studies at the National Institute of Radiological Sciences in Japan (17). They demonstrated favorable clinical outcomes compared to previous reports of X-ray RT and concluded that C-ion RT may constitute a promising alternative to surgical resection. However, these prospective clinical studies were performed at a single institution. Hence, we conducted a prospective observational study focusing on C-ion RT for treating pelvic recurrence of rectal cancer to confirm the reproducibility of clinical outcomes and to explore the benefits of this highly curative and less invasive local treatment strategy.

## Materials and Methods

### Patient Eligibility

Eligibility criteria for study participation were as follows: (1) the presence of pelvic recurrence of rectal cancer, as confirmed by histology or diagnostic imaging with computed tomography (CT), magnetic resonance imaging (MRI), and fluorodeoxyglucose positron emission tomography (FDG-PET); (2) the absence of distant metastasis; (3) patients had undergone curative resection for primary disease and regional lymph nodes, without gross or microscopic residual disease; (4) the presence of a radiographically measurable tumor; (5) Eastern Cooperative Oncology Group performance status ≤2; and (6) age 20–80 years. Patients were excluded if direct invasion of the bladder and/or intestinal tract was observed, if they had received chemotherapy and/or molecular targeted therapy within 4 weeks prior to the initiation of C-ion RT, if they had received prior RT to the target area, if they had intractable infections in the target area, if their tumor could not be covered by a field size of 15 × 15 cm, or if they had another active malignancy.

Pretreatment evaluation was performed before patient registration which included medical history, physical examinations, routine testing of blood cell counts, and chemistry, urine analysis, CT, MRI, and PET. Cystoscopy and/or proctoscopy were performed to exclude bladder or intestinal invasion when indicated. The treatment protocol for the current study was reviewed and approved by the Gunma University Institutional Review Board, and all patients signed an informed consent form before the initiation of C-ion RT. This study was registered at the University Hospital Medical Information Network in Japan (UMIN000009719, prospectively registered on January 8, 2013).

### Carbon-Ion Radiotherapy

C-ion beams were generated by a synchrotron at Gunma University Heavy Ion Medical Center. The passive scattering technique was applied for the treatment of pelvic recurrence of rectal cancer, and the beam energy was selected according to tumor depth; the available energies were 290, 380, and 400 MeV/u. At our facility, the radiation dose is calculated using XiO-N, which is an XiO (Elekta)-based software incorporating a dose engine for ion-beam RT (K2dose) developed by the National Institute of Radiological Sciences, with interfaces from Mitsubishi Electric (18). The C-ion RT dose was expressed in Gy [relative biological effectiveness (RBE)] which was defined as the physical dose multiplied by the RBE of carbon ions (19). Before C-ion RT, patients were immobilized using tailor-made fixation cushions and thermoplastic shells to acquire treatment planning CT images; respiratory-gated and 4-dimensional CT images were then acquired. Images from the expiratory phase were used for treatment planning. Patients received C-ion RT once daily, 4 days a week (Tuesday to Friday).

### Target Delineation and Treatment Planning

**Figure 1:**
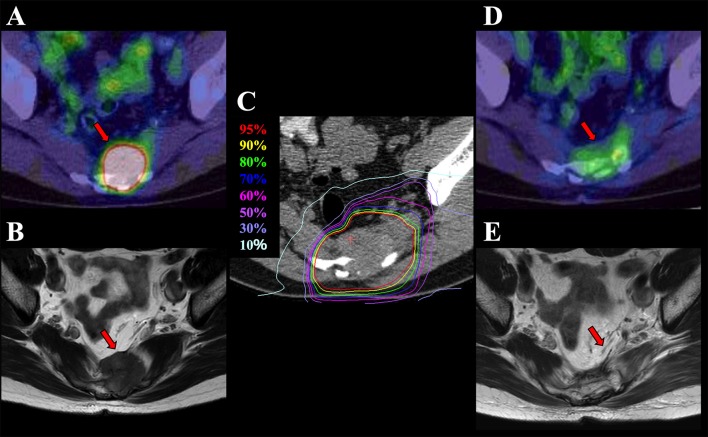
Pelvic recurrence of rectal cancer in a 58-year-old man treated with C-ion RT. **(A)** PET before treatment. **(B)** MRI before treatment. **(C)** Dose distribution on axial CT images. Highlighted are: 95% (red), 90% (yellow), 80% (green), 70% (blue), 60% (pink), 50% (purple), 30% (light purple), and 10% (light blue) isodose curves [100% = 73.6 Gy [RBE]]. **(D)** PET 3 months after treatment. **(E)** MRI 12 months after treatment demonstrating disappearance of the presacral mass. Arrows show the recurrent tumor. C-ion RT, carbon-ion radiotherapy; PET, positron emission tomography; MRI, magnetic resonance imaging; CT, computed tomography; RBE, relative biological effectiveness.

The treatment planning CT images were merged with the MRI and/or PET images to precisely delineate the gross tumor volume. The clinical target volume had a margin of least a 5-mm around the gross tumor volume to include microscopic disease. The internal margin was assessed with reference to 4-dimensional CT images for tumor movement. The planning target volume was defined as a summation of the clinical target volume, internal margin, and setup margin. Patient position was verified using digital orthogonal X-ray images and reference images that were digitally reconstructed based on the planning CT for daily patient position matching. C-ion RT was performed with 73.6 Gy (RBE) administered in 16 fractions over 4 weeks based on the schedule of the phase I/II and II studies at the National Institute of Radiological Sciences. The dose constraints were defined as the mean dose (Dmean) <50 Gy (RBE) and the maximum dose (Dmax) <60 Gy (RBE) to the intestinal tract and the dose delivered to a 1-cm^3^ volume of the bladder (D_1cc_) <60 Gy (RBE). Figure 1 shows a representative case of the dose distribution and diagnostic imaging in a case of pelvic recurrence of rectal cancer before and after C-ion RT.

### Evaluation During Follow-Up

Patients were followed-up for 1 month after C-ion RT completion and every 3 months thereafter. The follow-up examinations comprised routine testing of blood cell counts and chemistry and diagnostic imaging using CT, MRI, or PET. Acute and late toxicities were graded by the Common Terminology Criteria for Adverse Events, version 4.0 of the National Cancer Institute (20). Acute and late toxicities were evaluated as the highest grade of toxicity that occurred within 3 months and after 3 months, respectively, from the initiation of C-ion RT. Local recurrence was defined as the presence of tumor regrowth on CT, MRI, or PET in the irradiated tumor bed, with or without a continuous elevation of blood levels of tumor markers which included carcinoembryonic antigen and carbohydrate antigen 19-9. Regional recurrence was defined as marginal recurrence in the C-ion RT field or recurrence in the site of the original tumor or the para-aortic or contralateral pelvic regions.

### Statistical Analysis

All statistical analyses were performed using the JMP Pro 12.2.0 software package (SAS Institute, Inc., Cary, NC, USA). OS was measured from the date of initiation of C-ion RT to the date of death or most recent follow-up. Local control (LC) was defined as no evidence of local progression with no increase in tumor size on CT or MRI and no increase in FDG uptake on PET. Progression-free survival (PFS) was defined as no progression of both locoregional and distant metastases. PFS was measured from the date of initiation of C-ion RT to the date of observation of tumor progression or death from any cause. The probabilities of OS, LC, and PFS were calculated using the Kaplan-Meier method. The primary endpoint was the 3-year LC rate, and the secondary endpoints were the rates of OS, PFS, and acute and late toxicities. The variable risk was expressed as a hazard ratio with its corresponding 95% confidence interval (CI).

## Results

### Patient Characteristics

A total of 28 patients were enrolled between October 2011 and July 2017; the patient characteristics are summarized in Table 1. The median follow-up duration was 38.9 months (range: 7.6–74.1 months). The median age at the time of registration for C-ion RT was 63 years (range: 40–76 years). The median tumor size was 44 mm in diameter (range: 16–84 mm). A total of 24 and 4 patients were ineligible to undergo surgery and refused surgery, respectively. Direct invasion of the pelvic bone was observed in four patients. In terms of prior anticancer drug therapy, 16 patients had undergone adjuvant therapy after surgery, of whom 2 had undergone molecular targeted therapy combined with cytotoxic chemotherapy; 14 received cytotoxic chemotherapy alone. A total of 3 patients had received treatment after the recurrence of rectal cancer; 2 of them had received molecular targeted therapy combined with cytotoxic chemotherapy, and 1 had received cytotoxic chemotherapy alone. All patients received 73.6 Gy (RBE) in 16 fractions and completed C-ion RT as scheduled.

**Table 1:** Patient characteristics (*n* = 28).

**Characteristics**	
Age, years, median (range)	63 (40–76)
**PS, number**	
0	12 (42.9%)
1	16 (57.1%)
**Sex, number**	
Male	16 (57.1%)
Female	12 (42.9%)
**Primary tumor surgery, number**	
Abdominoperineal excision	16 (57.1%)
Low anterior resection	9 (32.1%)
Hartmann's resection	1 (3.6%)
Intersphincteric resection	2 (7.2%)
**Histology, number**	
Well-differentiated adenocarcinoma	10 (35.7%)
Moderately differentiated adenocarcinoma	15 (53.6%)
Mucinous adenocarcinoma	3 (10.7%)
Duration of surgery to C-ion RT, months, median (range)	25.6 (2.6–117.6)
**Tumor site, number**	
Presacral	7 (25.0%)
Side wall	17 (60.7%)
Perineal	4 (14.3%)
Tumor size, mm, median (range)	44 (16–84)
Serum CEA level before C-ion RT, ng/mL, median (range)	10.7 (0.3–617.3)

*CEA, carcinoembryonic antigen; C-ion RT, carbon ion radiotherapy; PS, performance status*.

### Overall Survival, Local Control, and Progression-Free Survival

**Figure 2:**
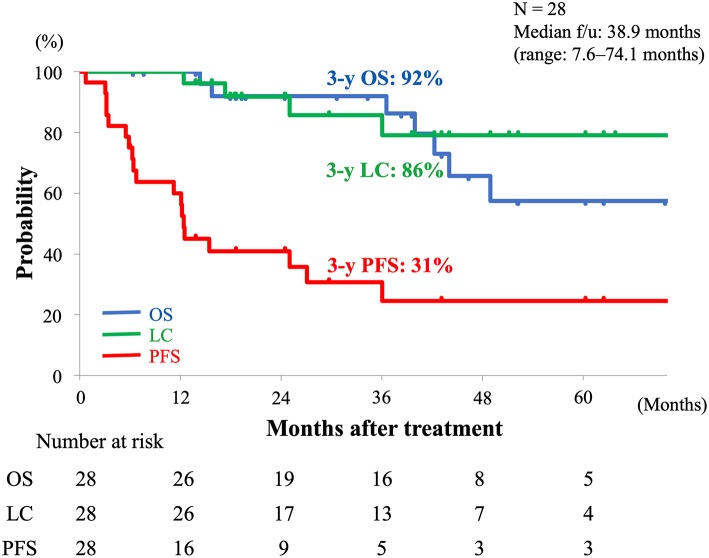
Kaplan-Meier curves of OS (blue line), LC (green line), and PFS (red line) of all patients. Number at risk is shown below the figure. OS, overall survival; LC, local control; PFS, progression-free survival.

The OS, LC, and PFS curves of all the patients are shown in Figure 2. The 3-year estimated OS, LC, and PFS rates were 92% (95% CI, 73–98%), 86% (95% CI, 63–95%), and 31% (95% CI, 16–52%), respectively. At the time of the analysis, recurrence after C-ion RT was observed in 19 patients; 4 had local recurrence, and 9 each had regional recurrence and distant metastases. Among the 9 patients with distant metastases, 3 had both locoregional and distant metastases. A total of 7 patients died of rectal cancer.

### Management of Post-carbon-Ion Radiotherapy Recurrences

Eighteen of 19 patients underwent salvage treatment for recurrence after C-ion RT. In terms of local recurrence, 3 patients received C-ion RT, and 1 received X-ray stereotactic body RT (SBRT). In the patients with regional recurrence alone, 2 received C-ion RT, 3 received molecular targeted therapy combined with cytotoxic chemotherapy, and 1 received cytotoxic chemotherapy alone. Among the patients with distant metastases with or without regional recurrence, 4 received molecular targeted therapy combined with cytotoxic chemotherapy, 1 received molecular targeted therapy alone, 2 received cytotoxic chemotherapy alone, and 1 underwent surgery.

### Toxicity

All the observed acute and late toxicities are listed in Table 2. In terms of acute toxicity, none of the patients developed grade 3 or higher hematological toxicities, and only 1 patient developed grade 2 radiation dermatitis. As for late toxicities, 2 patients developed grade 3 pelvic infections. One of these patients was a 62-year-old man with presacral recurrence of rectal cancer with a history of pelvic abscess at the recurrent tumor site before C-ion RT. He developed a pelvic abscess in the irradiated area 6 months after the initiation of C-ion RT, requiring drainage and intravenous antibiotics. Another patient was a 69-year-old man who experienced a perineal recurrence of rectal cancer. He developed a pelvic abscess in the irradiated area 17 months after the initiation of C-ion RT. He had received molecular targeted therapy combined with cytotoxic chemotherapy for distant metastasis before he developed the pelvic abscess. A total of 7 patients developed grade 2 peripheral neuropathy, of whom 3 had peripheral neuropathy before the initiation of C-ion RT.

**Table 2:** Acute and late toxicities graded by CTCAE, version 4.0 (*n* = 28).

	**Grade 0**	**Grade 1**	**Grade 2**	**Grade 3**	**Grade 4**
**Acute hematological toxicities**					
Leukopenia	25	2	1	0	0
Anemia	25	3	0	0	0
Thrombocytopenia	23	5	0	0	0
**Acute non-hematological toxicities**					
Dermatitis	17	10	1	0	0
GI tract	27	1	0	0	0
Urinary	26	2	0	0	0
Neuropathy	27	1	0	0	0
Infection	28	0	0	0	0
**Late non-hematological toxicities**					
Dermatitis	22	6	0	0	0
GI tract	27	1	0	0	0
Urinary	28	0	0	0	0
Neuropathy	15	6	7	0	0
Infection	26	0	0	2	0

*CTCAE, Common Terminology Criteria for Adverse Events; GI, gastrointestinal tract*.

## Discussion

The present prospective observational study was performed to confirm the reproducibility of C-ion RT and explore its efficacy as a highly curative and less invasive local treatment strategy for pelvic recurrences of rectal cancer. In our study, the 3-year OS, LC, and PFS rates were 92, 86, and 31%, respectively. In previous single-institution prospective and multi-institution retrospective studies using C-ion RT for pelvic recurrence of rectal cancer, the 3-year OS rates were 78 and 73%, respectively, and the 5-year LC rates were 88% for both (17, 21). Our study showed comparable clinical outcomes to the previous studies using similar therapeutic schedules in terms of dose fractionation and target volumes.

The most common curative treatment modality for pelvic recurrences of rectal cancer is surgical resection. Several researchers have reported that the 5-year OS rate after surgery in patients with pelvic recurrence of rectal cancer ranged from 15 to 60% (22–26). In terms of toxicities, however, Pereira et al. in their review reported that 7% to 50% of the patients developed pelvic abscesses, 5–10% developed intestinal obstructions, and 4–24% developed enterocutaneous or enteroperineal fistulas and secondary perineal wound dehiscence (26). Rahbari et al. reported that 42% of patients developed perioperative surgical morbidity with an in-hospital mortality rate of 3% (24). In contrast, our study demonstrated that complications after C-ion RT were relatively less, with 7% of the patients developing grade 3 pelvic infections and none developing intestinal obstruction. Although most of our participants were inoperable, the results, particularly in terms of OS after C-ion RT, were at least comparable to that of surgical resection. However, C-ion RT is less invasive than surgery.

X-ray RT was previously employed in treating inoperable patients with recurrence of rectal cancer, predominantly with palliative intent. However, combined with chemotherapy and recent advances in radiotherapeutic technology, it is being used to treat pelvic recurrence of rectal cancer with radical intent. Several researchers have reported the clinical outcomes of X-ray RT for recurrence of rectal cancer (27–33). Tanaka et al. reported the clinical outcomes after radical X-ray RT for pelvic recurrence of rectal cancer (33). In the study, the 1- and 3-year OS rates and the 1- and 3-year LC rates were 83 and 45%, and 52 and 20%, respectively. With respect to the relationship between dose and clinical efficacy, the 1-year OS and LC rates in the 75 Gy or higher biological effective dose (BED) group with α/β = 10 Gy (≥75 Gy_10_) vs. the 75 Gy or lower BED group (<75 Gy_10_) were 100 and 78% (*p* = 0.07) vs. 83 and 42% (*p* < 0.05). These results demonstrated that a higher BED may help in the achievement of favorable LC, potentially contributing to a better OS.

In recent years, X-ray SBRT, which can deliver a high dose to the target with high precision, has been widely used, and its clinical outcomes have been reported (28, 31). Franzese et al. demonstrated the clinical outcomes of X-ray SBRT for loco-regional recurrence of colorectal cancer with a median dose of 78.8 Gy_10_ (28). They showed that the 3-year OS and LC rates were 81 and 75%, respectively. Theoretically, C-ion RT can deliver a higher dose to the target volume than X-ray RT even if critical organs are nearby. In our study, the calculated C-ion RT BED was 107.5 Gy_10_, and regarding the clinical results, the OS and LC achieved by C-ion RT were more favorable than those achieved by X-ray RT and X-ray SBRT (Table 3). The present study showed that the use of a higher BED leads to favorable LC which may contribute to a better OS.

**Table 3:** Comparison of the present study with previous studies on recurrence of rectal cancer.

**References**	**Year**	***n***	**Method of treatment**	**3-year OS rate**	**5-year OS rate**	**3-year LC rate**	**5-year LC rate**	**Late toxicities ≥grade 3**
Wong et al. (6)	1998	214	X-ray RT	NA	5%	NA	7%	Infection: none, GI: 5.7%
Tanaka et al. (33)	2017	32	X-ray RT	45%	23%	19%	13%	NA
Lee et al. (32)	2011	22	CCRT	NA	41%	NA	56%	NA
Cai et al. (27)	2015	71	CCRT	37%	NA	34%	NA	NA
Kim et al. (31)	2008	23	SBRT	NA	23%	74% (4-year)	NA	Infection: none, GI: none
Franzese et al. (28)	2017	35	SBRT	81%	NA	75%	NA	Infection: none, GI: none
Yamada et al. (17)	2016	151	C-ion RT	78%	59%	NA	88%	Infection: none, GI: 0.7%
Shinoto et al. (21)	2018	224	C-ion RT	73%	51%	93%	88%	Infection: 3.1%, GI: 0.9%
Present study		28	C-ion RT	92%	58%	86%	79%	Infection: 7%, GI: none

*CCRT, concurrent chemoradiotherapy; C-ion RT, carbon ion radiotherapy; GI, gastrointestinal tract; LC, local control; NA, not available; OS, overall survival; RT, radiotherapy; SBRT, stereotactic body radiotherapy*.

Systemic therapy has an important role in improving the prognosis of pelvic recurrences of rectal cancer. A previous study showed that OS was improved in the group undergoing X-ray RT with chemotherapy compared with that of the X-ray RT-alone group (29). Since regional recurrence and/or distant metastases occurred after C-ion RT in our study, the PFS rate was unsatisfactory. Concurrent or adjuvant use of chemotherapy may lead to improved PFS and OS with C-ion RT for pelvic recurrence of rectal cancer.

Recently, immunotherapy is an important topic in terms of anticancer treatment. Sato et al. reported an *in vitro* study in which DNA double-strand breaks as a result of X-ray irradiation upregulated PD-L1 expression in human osteosarcoma, lung cancer, and prostate cancer cell lines (34). Additionally, Oike et al. reported an *in vitro* study of human uterine cervical cancer in which radiotherapy-induced double-strand breaks were related to linear energy transfer, and double-strand breaks were observed more frequently with C-ion beam irradiation than with X-ray irradiation (35). Although there were no data of C-ion beam irradiation upregulating PD-L1 expression, the high linear energy transfer of C-ion RT has a potentially synergistic effect with immunotherapy using anti-PD-1 and anti-PD-L1 immune checkpoint antibodies, because C-ion RT could induce more double-strand breaks than X-ray RT and, C-ion RT combined with immunotherapy has potentially improved PFS and OS.

In the present study, grade 3 late toxicities were observed in 2 (7%) of the 28 patients; both developed grade 3 pelvic infections in the irradiated area. The presence of a past history of abscess and neutropenia due to chemotherapy may have contributed to the development of the pelvic abscesses. In previous studies on C-ion RT for pelvic recurrence of rectal cancer, 2 and 5% of patients developed grade 3 late toxicities (18, 22). The incidence of late toxicities in the present study was comparable to that observed in previous reports on C-ion RT.

Our study has some limitations. First, this prospective study was conducted at a single institution. A multi-institution prospective study is currently underway which will elucidate the efficacy and toxicity of C-ion RT in treating pelvic recurrence of rectal cancer. Second, patients with direct invasion of the bowel/bladder were excluded because of the high risk of severe toxicities such as perforation in this prospective study. Therefore, the toxicity might not be truly representative. Third, although this study showed favorable clinical outcomes, the follow-up duration was too short to determine the long-term safety of this treatment.

In conclusion, C-ion RT showed favorable OS and LC with low rates of toxicity in treating pelvic recurrence of rectal cancer. Our study demonstrated superior outcomes of C-ion RT for pelvic recurrence of rectal cancer, suggesting a potential role for C-ion RT in the radical treatment of patients who are unsuitable for surgery.

## Data Availability

All datasets generated for this study are included in the manuscript.

## Ethics Statement

The studies involving human participants were reviewed and approved by Gunma University Institutional Review Board. The ethics committee waived the requirement of written informed consent for participation.

## Author Contributions

SS, MO, HKi, TO, and TN made substantial contributions to the conception and design of the study. SS, MO, HKi, TK, and TO treated and followed up the patients. SS, MO, and HKi collected the data. SS, MO, and TO drafted the manuscript and performed the statistical analysis. TO, TA, HO, KS, HKu, and TN were involved in critically revising the manuscript for important intellectual content. SS, MO, and TO participated in the acquisition and interpretation of the data. All authors read and approved the final manuscript.

### Conflict of Interest Statement

The authors declare that the research was conducted in the absence of any commercial or financial relationships that could be construed as a potential conflict of interest.
